# Hybrid Feature Selection Framework for the Parkinson Imbalanced Dataset Prediction Problem

**DOI:** 10.3390/medicina57111217

**Published:** 2021-11-08

**Authors:** Hayder Mohammed Qasim, Oguz Ata, Mohammad Azam Ansari, Mohammad N. Alomary, Saad Alghamdi, Mazen Almehmadi

**Affiliations:** 1 Department of Electrical and Computer Engineering, Institute of Science, Altinbas University, Istanbul 34218, Turkey; oguzata@gmail.com; 2 Department of Epidemic Disease Research, Institute for Research & Medical Consultations (IRMC), Imam Abdulrahman Bin Faisal University, Dammam 31441, Saudi Arabia; 3 National Centre for Biotechnology, King Abdulaziz City for Science and Technology (KACST), Riyadh 11442, Saudi Arabia; malomary@kacst.edu.sa; 4 Laboratory Medicine Department, Faculty of Applied Medical Sciences, Umm Al-Qura University, Makkah 24382, Saudi Arabia; ssalghamdi@uqu.edu.sa; 5 Department of Clinical Laboratory Sciences, College of Applied Medical Sciences, Taif University, Taif 21944, Saudi Arabia; Dr.mazen.ma@gmail.com

**Keywords:** Parkinson detection, machine learning, PCA, RFE, SMOTE

## Abstract

*Background and Objectives*: Recently, many studies have focused on the early detection of Parkinson’s disease (PD). This disease belongs to a group of neurological problems that immediately affect brain cells and influence the movement, hearing, and various cognitive functions. Medical data sets are often not equally distributed in their classes and this gives a bias in the classification of patients. We performed a Hybrid feature selection framework that can deal with imbalanced datasets like PD. Use the SOMTE algorithm to deal with unbalanced datasets. Removing the contradiction from the features in the dataset and decrease the processing time by using Recursive Feature Elimination (RFE), and Principle Component Analysis (PCA). *Materials and Methods*: PD acoustic datasets and the characteristics of control subjects were used to construct classification models such as Bagging, K-nearest neighbour (KNN), multilayer perceptron, and the support vector machine (SVM). In the prepressing stage, the synthetic minority over-sampling technique (SMOTE) with two-feature selection RFE and PCA were used. The PD dataset comprises a large difference between the numbers of the infected and uninfected patients, which causes the classification bias problem. Therefore, SMOTE was used to resolve this problem. *Results*: For model evaluation, the train–test split technique was used for the experiment. All the models were Grid-search tuned, the evaluation results of the SVM model showed the highest accuracy of 98.2%, and the KNN model exhibited the highest specificity of 99%. *Conclusions*: the proposed method is compared with the current modern methods of detecting Parkinson’s disease and other methods for medical diseases, it was noted that our developed system could treat data bias and reach a high prediction of PD and this can be beneficial for health organizations to properly prioritize assets.

## 1. Introduction

Parkinson’s disease (PD), a condition that affects the elderly more than people at other ages, comes after Alzheimer’s disease and results from neurological disorders [1,2]. In 2015, a study reported that 177,000 people died from PD [3] because of the decrease in correct diagnostic treatment, which led to numerous PD cases. PD occurs as an outcome of the loss of dark matter with age in dopaminergic neurons. For PD, one of the optimum treatment options available is clinical monitoring for delayed dopamine loss. Therefore, diagnosing and detecting the illness in its early stage is most suitable. In addition, early detection by measuring the sound signals of Parkinsons patients helps diagnose PD years before the onset of clinical symptoms [4]. Although no definitive treatment has been reported to eliminate this disease, with advancement of science, researchers have used various approaches to combat it. Moreover, with the help of different branches of science, much progress has been made in controlling PD [5,6]. An emerging technology that helps clinicians in the early diagnosis and treatment of a disease is machine learning (ML). ML methods can be satisfactorily used to classify diseases and that a rating system allows accuracy enhancement, analysis reliability, and error reduction, making the system highly efficient [7].

In addition, numerous soft computing methods have been proposed in studies on predicting PD for enhancing the prediction accuracy and reducing the error rate. These technologies include deep learning (DL) [8], genetic algorithm (GA) [9], and XGBoost method [10]. A study used an ensemble learning classifier, which can provide a high prediction accuracy [11]. Although considerable efforts have been taken to make models highly accurate for PD prediction, the task remains challenging due to numerous reasons. First, many variables can be directly or circuitously affected by classification models. Because most medical datasets can be unbalanced, the number of uninfected patients is much greater than that for the number of infected patients, which leads to a prediction bias. Furthermore, a large distribution of features causes noise and an increase in the pre-processing time. All these problems can cause a considerable loss of the detection accuracy.

This paper addresses the aforementioned problems to provide a highly accurate prediction system by identifying the main factors affecting PD. We used a complex method of PD prediction that relies on three major steps: balancing, feature selection, and model optimisation. In the data balancing step, unbalanced class allocation was treated using the synthetic minority oversampling technique (SMOTE).

Pramanik et al. [11] used enhanced decision forest algorithms using systematically developed forest (SysFor), with penalising attributes (ForestPA), and public random forest algorithms and compared the results for two groups of recent acoustic data on PD. Decision forest with penalising attributes is the optimum solution for detecting PD with an accuracy of 94.12–95%. Another study proposed an improved RAO algorithm with an enhanced KNN classifier as a *k* parameter value for solving optimisation problems applied to four datasets of patient with PD [12]. In addition to selecting the optimum set of features, the results proved that revised treatment can highly assort PD. Borzì et al. proposed a method for predicting and managing signals to prevent the patients with PD from gait freezing by applying ML algorithms to the data of a group of the patients with PD and improving the weaknesses of their system. The proposed algorithm achieved good results for patient appearance. Inside and outside the treatment, the final results of the system indicated an accuracy rate of 87.4% [13]. Quan et al. [14] suggested the use of long–short-term memory(LSTM) to detect vocal changes in the patients with PD to intervene in rapid treatment before the patients’ physical complications lead to disability. The processing method involves dividing of the audio data into two groups. The results showed an improved accuracy after combining ML with static features.

Raza et al. [15] presented a model that uses ML with the Internet of things(IoT) to follow auditory inputs by analysing patients’ environmental conditions as well as managed and followed up with priority contacts to discover how PD progresses within six months. The results showed an efficient communication schedule for many users with low latency. Beruset et al. [16] used multiple frontal feeding neural networks to predict PD. Many treatments were used to select numerous features established on the Pearson correlation coefficient, the Kendall correlation coefficient, and number of factors contributing to the performance of the proposed algorithm, and the optimal result on voice samples for the patients with PD was obtained by selecting the Kendall correlation coefficient. The optimal rating result reached approximately 86% accuracy. Mishra et al. [17] used an enhanced adaptive-genetic algorithm (EAGA) to identify the main risk factors for diabetes, which can help the health staff to classify this condition accurately. Moreover, their results showed the effectiveness of applying their technique to seven types of diabetes. Kaur et al. [18] employed deep learning to classify healthy people and people with PD. In their method, the optimal features were separated from all the major features, and then deep learning was used to classify the vocal pitch between healthy people and people with PD. 

Studies have reported that the effectiveness of a PD detection system mainly depends on the enhancement of classifier recognition and accuracy. However, the problems of unbalanced data, noise, and redundant features are not effectively optimised. Hence, these problems can cause a considerable loss in the detection accuracy. Moreover, the result of this study can indicate the potential for improving PD detection system with several ML models. 

## 2. Methodology

The methodology used to solve the problems in the PD dataset is described. The principal steps involved in the proposed methodology are to develop a highly efficient ML system to enhance imbalance datasets. Our proposed methodology includes five phases:

(1) Pre-processing of a PD dataset,
(2) splitting,
(3) theoretical contexts of several ML techniques,
(4) hyper-parameter settings, and,
(5) evaluation of ML performance across different metrics.

**Figure 1:**
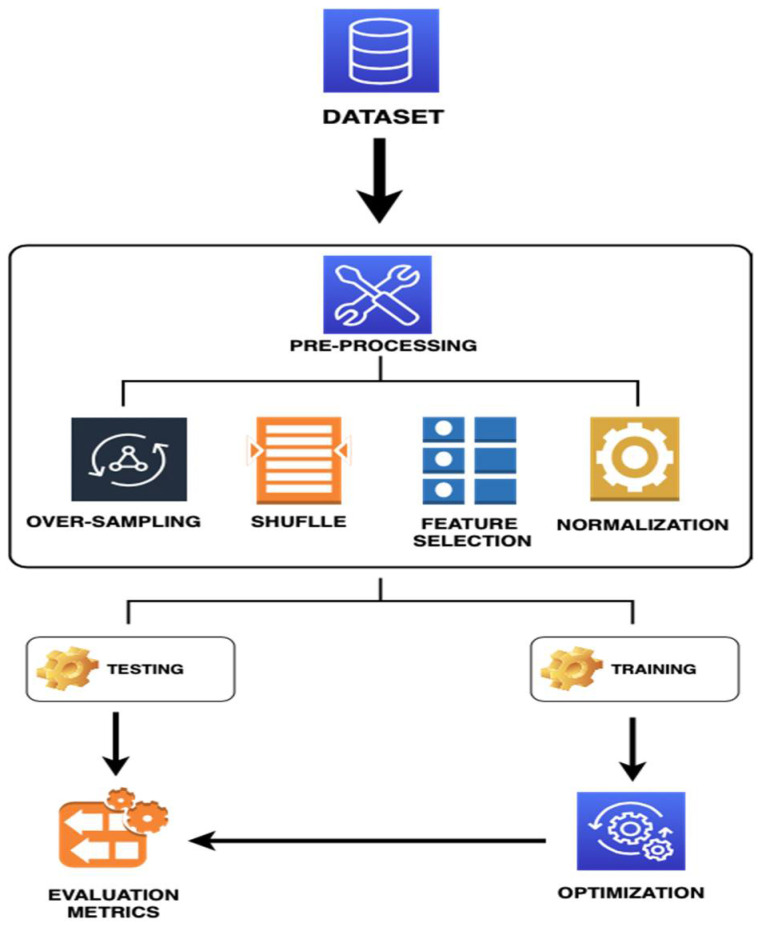
Flowchart of the Machine Learning for PD Prediction System.

The methodology is illustrated in Figure 1 and described in the next subsection.

### 2.1. Data Description

The PD dataset comprised 756 samples and 753 features. This dataset can be accessed by the UC Irvine Machine Learning Repository [19]. The PD features were obtained by processing speech signals. Moreover, the PD data were acquired from 188 patients PD (107 men and 81 women) with age of 33–87. In general, basic features are frequently used and recognised by technicians involved in PD diagnosis. Specialised software is used to extract the features from the auditory stream. In this study, we investigated both the primary and derived features. Due to a high proportion of the patients with PD, the dataset is class-imbalanced. In the dataset, 14 primary feature merits and four derived feature merits exist Table 1. The technicians who frequently diagnose PD used this dataset to recognise primary features. By contrast, the dataset presents two problems: the high-distribution features, and the unbalanced data because there are 192 healthy patients and 564 patients with PD. 

Differences in the original data between positive and negative patients for a PD dataset are presented in Table 2. The balanced data were obtained by increasing the minority samples into the original data by using SMOTE. The classes completely balanced after over sampling with 564 positive and 564 negative cases.

### 2.2. Dataset Preprocessing

Data pre-processing involves a set of suboperations comprising several procedures that can be applied to datasets for analysis and formation. Thus, data pre-processing is a vital step in the data mining system [20]. In this study, data pre-processing procedure involved data cleaning, organisation, and resampling. In data cleaning, inconsistencies and the noise (i.e., incorrect data access) were eliminated. Then, the SMOTE method was used to resolve the problem of the imbalanced data by randomly producing new samples in minority group samples and their neighbours. Data shuffling was performed to reduce variability and ensure that the models remained generic and less overfit. Feature selection is another ML technique, in which input (attribute) diversity is reduced to identify most vital features for model building. Hence, the information normalisation approach was used to convert all the variable values into a special range to obtain a strong relationship between them. 

### 2.3. Re-Sampling with SMOTE

The common problem with medical data is imbalance because the number distribution of patients is lower than that of the control group, which leads to a problem in ML classification. In our dataset, the ratio of non-patients has the large majority in classes, which can lead to bias in the classification accuracy towards only the non-patients. To resolve this problem, SOMTE is a powerful ML technique with the key concept of randomly generating new samples among minority group samples and their neighbours, which increases the number of minority group samples and causes the class to be balanced [21]. SMOTE, first proposed in Chawla et al. [22], is a great example of the pattern augmentation technique. SMOTE is used to generate new samples for the minority class [23] by taking a pattern from each minority class and creating new synthetic samples along with a group in the class and a minority in each of its closest, randomly identified neighbour [24].

### 2.4. Feature Selection

Selecting the features that are applicable or most important to the classification problems is critical to determine the categories required. Thus, PD classification is complex because of which there is an expansion of needless or unrelated features. If the entire dataset containing numerous features is used, calculations become laborious and time consuming. Therefore, in this study, the future selection technique was applied as an important process in the pre-processing stage. Hence, the number of features must be minimised to obtain the most important features useful for the initial step in the learning procedure of the model, which leads to an easy understanding of the results and an increase in the classification accuracy, while simultaneously improving the overall performance of the model. Two methods are used for feature selection and are described in the following subsection.

#### 2.4.1. Recursive Feature Elimination (RFE)

The PD dataset contains 752 features; if all these features are used in training, the processing time increases and predictability decreases. Furthermore, the problems such as duplicate features, ineffective features, and great contrast between features can be confronted [25]. To resolve all these problems, RFE is used with the cross-validation technique to obtain a subset of the most applicable features to prediction problems. The model works within the framework of the wrapper method to ensure that the most impactful subset of features is acquired. Therefore, using this model is critical for identifying features of the large or small datasets.

We can interpret the implementation of the RFE as follows:

Step 1. Construct a feature set with function vectors so that each row matches to a sample, and each column matched to an attribute.

Step 2. Use control parameters to create the RFE method with 10-fold validation.

Step 3. Use RFE to sort these features according to their relation with the target.

Step 4. Based on the results for the highest impact features, select the first N (number of features) with the highest correlation with the target as the new feature of the data, and remove the lowest correlation with the target from the data.

#### 2.4.2. Principle Component Analysis (PCA)

PCA is primarily based on a statistical–mathematical approach that uses an eigenvector for minimising the size of a record set, which includes more than one variable associated with different retaining and the highest variance inside the dataset [26,27]. PCA is considered a statistical technique, which depends on its orthogonal components of linear dataset, whose first part includes a set of variables and linear data that mostly comprises the information related to variables. Because the score is calculated and measured in a range from 0 to 100, the higher is the score, the greater is the quality. The dataset used in PCA should be a scaled set, and the method summarises the effects of producing statistics, which can be additionally sensitive to a proportional measurement. PCA evaluation maps the n-size feature area into k-dimension, and the covariance matrix is calculated. Subsequently, the calculated result is used to determine the eigenvectors and eigenvalues [28]. The main component is the eigenvector with the highest eigenvalue and is used to define the most important relationship between record set attributes in the PD data.

Furthermore, in PCA, the dimensional reduction concept is utilised for image compression, computer vision, and facial recognition. It also presents a wide range of applications in determining the high-dimensional record patterns related to data mining, finance, psychology, and bioinformatics [27,28,29,30].

### 2.5. Normalizing

The feature values in the PD dataset have different ranges, which leads to noise in classification performance. Therefore, normalisation is performed on the dataset [30] to set the data in a uniform range of [0,1]. Normalisation is calculated using the following Equation:

(1) $$f_{i}^{\mathrm{new}}=\frac{f_{i}^{\mathrm{old}}-\min\left(F\right)}{\max\left(F\right)-\min\left(F\right)}$$

where $f_{i}\;$ represents the current value of the feature, and min(F) and max(F) denote the minimum and maximum values of the feature, respectively.

### 2.6. Machine Learning Classification Algorithm

In this Section, the theoretical contexts of the four ML classification techniques are described and explained.

Support vector machine (SVM) can be defined as a supervised learning method, whose working mechanism depends on classification, external detection, and regression by training data; it can also be used to solve binary and multiple classification problems. Moreover, a task assigned to this algorithm is to solve the problems of linear and nonlinear data. For the training data, it is used to separate the data into two groups with high and low dimensions, and the gap between the two groups is called the hyperplane. The optimal hyperplane can measure the space between two dimensional by using two equations (W^t^ X + b = +1) for high dimensional and (W^t^ X + b = −1) for low dimensional. The supper vector is the data on the separate line when the line is the decision boundary [31].

Multilayer perceptron (MLP) is an algorithm of the neural network character, which shows flexibility, reliability, and nonlinearity in performance. This algorithm comprises three layers of neural networks; the first layer enters the primary data and transfers the data to the second layer, which is called the hidden layer. This layer processes and sends the data to the last layer that provides the final prediction. A benefit of this algorithm is that it can adapt itself and learn from the nonlinear data. Moreover, it can predict the invisible data and large databases and provide satisfactory results [17].

Bagging is an ensemble method with a strong and effective character in learning. This method uses a set of algorithms by learning, and afterwards, its work is classified. The packing method faces two challenges during its main operation:

(1) In this method, one of the advantages of bootstraps slightly varies, unlike in other assembly methods.
(2) In the bootstraps, the number of bootstraps cannot be counted.

This results in some extra classifiers that lead to a decrease in the classification speed, a decrease in the packing efficiency, and an increase in the memory capacity, which is different from other assembly methods [32].

K-nearest neighbours’ algorithm (K-NN) is a type of ML algorithm subject to supervision, classification, and pattern recognition. This algorithm relies on classification and efficiency accuracy of proximity and neighbour points. In addition, its internal structure is complex, and its ability to read is not strong. Its local geometry, dimensions, and quantum state resulting from the logical quantum line in the unspecified qubits are registered. Compared with the classical quantitative algorithm, k-NN can use an increased amount of data and a quantum property to store the data, thus reducing and enlarging the allocated data area [33].

### 2.7. Hyperparameter for Machine Learning Model

Each ML classification model requires one or more criteria, which manage (efficacy) the predictive consequences of the classifier. Selecting fine values for these parameters is highly challenging and requires the search of an exchange of among model generalisation and model complexity [20]. In grid search, the value depending on a range of model parameters is changed, and each parameter with a verified time interval is incremented before finding the optimal parameter values. In this study, optimisation was performed to obtain optimal model performance; thus, we conducted a grid search across a grid of selected parameters to obtain a set of the best-performing parameters. Furthermore, optimisation is used to reduce the prediction time, the error rate, and over-processing and to find the optimal hyperparameter.

### 2.8. Evaluation Metrics of HD Performance

The evaluation process can be used to assess the validity and accuracy of these constructed models acquired through the following classification counters:

True positive (TP) indicates the number of accurately rated positive samples.

False positive (FP) indicates the number of negative and correctly classified samples.

True negative (TN) denotes the number of positive and incorrectly classified samples.

False negative (FN) is the number of incorrectly classified samples for the negative class.

Four evaluation measures are used to assess the results of classifiers, which depend on the following four classification counters:

Accuracy: It is used to determine classifier effectiveness from the correct expected percentage, as shown in Equation (2).

(2) $$\mathrm{Accuracy}=\frac{\mathrm{TP}+\mathrm{TN}}{\mathrm{TP}+\mathrm{TN}+\mathrm{FP}+\;\mathrm{FN}}$$

Sensitivity: It is the percentage of the accurately classified positive samples with PD and is called as the TP rate (TPR). It can be calculated as shown in Equation (3).

(3) $$\mathrm{Sensitivity}\;=\frac{\mathrm{TP}}{\left(\mathrm{TP}\;+\;\mathrm{FN}\right)}$$

Specificity: It is the percentage of the negative samples without any correctly classified PD and is called as the TN rate (TNR). It is calculated as presented in Equation (4).

(4) $$\mathrm{Specificity}\;=\frac{\mathrm{TN}}{\left(\mathrm{TN}\;+\;\mathrm{FP}\right)}$$

Precision: It is an important metric for accuracy measurement and defines the percentage of cases, which are identified as positive by the classifier, for the relationship between total predictive positive states, as shown in Equation (5).

(5) $$\mathrm{Precision}=\frac{\mathrm{TP}}{\left(\mathrm{TP}+\mathrm{FP}\right)}$$

All these experimental measures are insufficient to rely on to assess learners within an unbalanced data set. Accuracy is a misleading evaluation metric for the majority class and seldom predicts the parameters belonging to the minority class. For the classification problems involving unbalanced class distribution, we seek highly comprehensive evaluation by considering various aspects comprising the measurement of the classifier ability to attain a balance between two classes and considering both classes to be similar [30]. In our experiments, the area under the curve (AUC) and geometric imply (G-mean) are used because they exhibit robustness for unbalanced data distribution within the data.

G-mean: It is a function used to filter the optimum classification between two classes. A benefit of this function is that it minimises the negative category when the false-positive results increase. Moreover, the arithmetic mean is considered an important measure for sensitivity and specificity determination [34]. Sensitivity is (TP/(TP + FN), and specificity is (TN/(TN + FP); therefore, G-mean is presented as Equation (6):

(6) $$G-\mathrm{mean}\;=\sqrt{(\mathrm{sensitivity}\times \mathrm{specifity}}$$

AUC is particularly used in binary classification to determine the optimum model for category prediction; in the AUC score, a threshold is employed to calculate the ratio of the TP rate and FP rate.

## 3. Results and Discussion

This section presents the results obtained using the PD datasets defined in Section 2.1. The PD datasets, such as medical datasets, have the problem of unbalanced datasets, and this problem leads to inaccurate classification and affects the results. Therefore, we applied a new system to train several ML classification models. In the preprocessing stage, the SMOTE over-sampling technique was employed to overcome the imbalanced dataset problem because the number of (nonpatient) minority class was increased by producing new synthetic samples. Thus, the problem of the unbalanced class was processed using the SMOTE algorithm in PD dataset classification, and two feature selection techniques, RFE with 10 cross-validation and PCA, were implemented to reduce the variety of features in the PD dataset. The ML methods used to diagnose whether patients have PD in this study included: MLP, SVM, K-NN, and Bagging; the examination was conducted by splitting the data into 80–20 in the test–train split approach. Normalisation was applied to all the features before they were implemented on classifiers.

Table 3 presents the overall performance of the dataset obtained before and after preprocessing implementation. Moreover, five evaluation metrics were used: accuracy, precision, sensitivity, specificity, and G-Mean. Each classifier used 80% data from dataset for training and 20% data for the classifier examination phase of learning. For the original dataset, the optimum evaluation performance was achieved using K-NN with an accuracy of 87.4%, 753 features, and 756 total patient records (605 and 151 records used for training and testing sets, respectively), and SVM was the second-best model with 84.3% accuracy (Table 3). After applying SMOTE, as the optimum model with RFE, MLP reached the accuracy of 93.3% with 329 features and 1128 patient records (902 and 256 records for training and testing). SVM was the second-best model with 90.2% accuracy. Moreover, RFE was applied with PCA to reduce the number of features from 329 to 18 and presented excellent evaluation results by examining all classifiers with 902 and 256 patients as the training and testing sets, respectively, and its best accuracy was 95.1% with MLP. For each sample classifier presented in Table 3, the models trained with RFE + PCA continuously produced the relatively higher accuracy with good development in classification performance.

Among the four considered classifiers, MLP and K-NN provided the highest and lowest accuracy rates, respectively. Consequently, the proposed method considerably influenced accuracy rate enhancement for the four classifiers. The overall performance results showed that the proposed technique can work successfully with various classification models. Table 3 presents the variations in the predictive accuracy estimates acquired from all the ML models of the Istanbul audio signal dataset. In this study, over-sampling and two feature selection were applied to the dataset as a preprocessing step. Moreover, the classification performance results of all the models were comprehensively discussed. Therefore, the proposed PD diagnostic system can help medical practitioners efficiently identify the patients with PD.

All the classifiers were implemented using default parameters provided in Table 3. Algorithms depend on the hyperparameter settings of the classifier to achieve optimal performance for the classification problem. We conducted grid search to tune the parameters to achieve the best performance for each classification algorithm (Table 4). Accuracy scores for all ML models reached a minimum of 93.8% (Table 5). The optimum-performance models were obtained using the SVM model with an accuracy of 98%. MLP and Bagging were referred to as the second- and third-best models, respectively.

**Figure 2:**
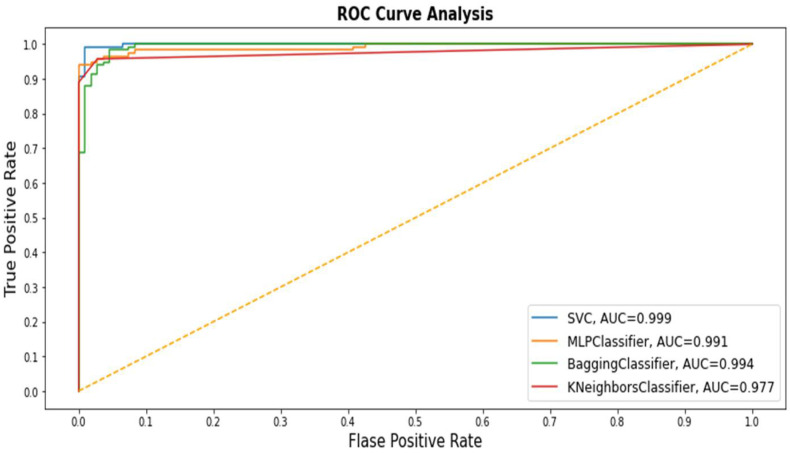
Receiver operating characteristic (ROC) values of all Machine Learning (ML) application models.

The ROC curve analysis or AUC is also a good evaluative measure for determining the performance of various class classification methods at the enter data stage. We adopted AUC to evaluate the performance of all classifiers. The ROC curve is plotted against TP and FP rates (Figure 2). The SVM curve appears with a blue line that depicts the optimal curve compared with other class curves. KNN performance was the lowest among the all methods for the ROC curve analysis. Thus, the AUC degree has a crucial function in medical research because it contains a meaningful explanation of classification of patients with disease from healthy people [35].

The approach proposed to detect PD provided convincing results for solving the unbalanced data problem and finding the most relevant features. However, it is exciting to compare our approach with recent studies. In this regard, few recent studies on PD detection techniques were compiled and compared with our study. Table 6 presents the comparison results between this study and recent studies. Convolutional neural network (CNN) [8] method reached 86% accuracy without reducing the features, whereas minimum mean maximum tree (MAMT) [36] obtained 92% accuracy with 50 features. The XGBoost [10] algorithm was also implemented to classify PD. Furthermore, random forest [11] was implemented an important ML algorithm with a decrease in the number of trees. All these methods are based on the Istanbul audio dataset used in this study. The results were compared with the same evaluation criteria used in this study. The comparative analysis is shown in Table 6.

Compared with the other studies, the optimum accuracy with few features was obtained using our proposed approach (Table 6). Thus, the proposed approach is superior to other detection methods for diagnosing PD by identifying the most important features.

The major limitation of this study is the small number of patients (107 men and 81 women) with a lack of the laboratory results. Within a measurable population range, the pre-diction accuracy of 87.4 was achieved. However, numerous variables can directly or circuitously affect the classification model. Because most medical datasets are unbalanced, the outcome for the number of the uninfected patients is considerably larger than that of the infected patients, which leads to a prediction bias. Furthermore, a large feature distribution causes noise and an increase in the pre-processing time; all these problems can result in a substantial loss in the detection accuracy. Therefore, the information was imbalanced; thus, we balanced it by using SMOTE. The performance of these models may be progressed by using relatively more data.

## 4. Conclusions

With the rapid development in the field of biomedicine, ML classification has played an increasingly fundamental role. ML models can help enhance the classification accuracy and evaluation reliability and reduce potential misunderstandings, in addition to making predictions highly efficient. In this study, two recent datasets for the patients with PD and control subjects were used to predict PD. A major problem with ML in the medical dataset is that the data collected is highly unbalanced, and thus additional capabilities are required to appropriately overcome bias distribution. To resolve this problem, several ML methods, such as SMOTE (over-sampling) technique, and two methods for feature selection, RFE and PCA, are used to predict PD. Moreover, advanced models have been evaluated for accuracy, precision, G-mean, sensitivity, and specificity. For classify positive or negative PD cases, supervised ML models were developed in this study with MLP, SVM, the Bagging classifier, and KNN. For the overall evaluation performance of the models, the train–test split approach was applied to the original and pre-processing data. The optimal evaluation metrics was obtained with the original data by using the KNN model with the accuracy of 87%. MLP with the pre-processing data reached both the accuracy and precision of 95%. Furthermore, the model developed with SVM optimisation was the optimal model among all the models developed for accuracy (98.22%). 

Future studies with other PD features obtained from other sites must be performed to validate these findings. We only analysed the samples from the Istanbul audio dataset. Moreover, the proposed approach improved the accuracy compared with the same dataset used in previous studies. Furthermore, this methodology was applied onto and evaluated on the small number of patients from the Istanbul audio dataset, which comprises the highly distributed and unbalanced data. In the future, we will concentrate on the use of other strategies, such as cost sensitivity, in combination with other methods as an alternative to resolve the imbalance problem of the medical dataset class.

In conclusion, the proposed method is compared with existing modern methods for PD detection and other approaches for medical diseases. Comparative analyses showed that the proposed method recognises patients with PD more effectively than its counterparts.

## Figures and Tables

**Table 1:** PD dataset from UCI.

Metrics	Features Clarification	Nº. Features
	**Primary Feature**	
Jitter variants	The uncontrolled vibration of the ropes causes jitter, which indicates the frequency change from one cycle to the next.	5
Shimmer	indicates a change in the amplitude of a sound wave.	6
Fundamental frequency parameters	The vibration of the vocal folds is represented by the frequency.	6
Harmonicity parameters	Noise components in speech arise due to partial closure of the vocal folds.	2
Recurrence Period Density Entropy	Provides evidence of the vocal folds’ ability to endure fluctuations in the fixed vocal folds, as well as the disparities between the two.	1
Detrended Fluctuation Analysis	This is used to assess the similarity of noise produced by turbulent airflow in the vocal folds.	1
Pitch Period Entropy	is a measure of fundamental frequency’s impure confusing impact. It calculates entropy and utilizes the logarithmic scale.	1
Intensity Parameters	The loudness of a spoken signal is measured by its intensity. It has something to do with the airflow’s subglottis strain.	3
Formant Frequencies	It’s utilized to test the vocal tract filer’s frequency response.	4
Bandwidth	Among the formant frequencies, the bandwidth frequency range.	4
Glottis Quotient (GQ)	It offers proof of the glottis’ opening and shutting intervals.	3
Glottal to Noise Excitation (GNE)	It quantifies the amount of chaotic noise caused by insufficient vocal fold closure.	6
Vocal Fold Excitation Ratio (VFER)	Using non-linear energy and entropy estimation, it calculates the volume of noise on the vocal fold vibration.	7
Empirical Mode Decomposition (EMD)	Adaptive basis functions are used to breakdown a voice signal into basic components, from which strength and entropy are computed.	6
	**Derived Feature**	
Mel-Frequency Cepstral Coefficients (MFCC)	Mel Frequency characteristics are calculated using the PD voice signal.	84
Wavelet transform (WT)	To determine the WT features set on the voice signal, Wavelet Transformation is used.	182
Tunable Q-Factor wavelet transform (TQWT)	The use of a configurable Q-factor to quantify deviations from fundamental frequencies	432

**Table 2:** Comparative between patients and non-patients samples.

PD Dataset	Positive PD	Negative PD	Total
Original	564	192	756
SMOTE	564	564	1128

**Table 3:** Classifier evaluation results with the original and preprocessed versions of the PD dataset.

Classifier	Prepressing	Accuracy	Precision	Sensitivity	Specificity	G-Mean
MLP	Original	82.2	0.82	0.78	0.71	0.755
	RFE	93.3	0.971	0.921	0.95	0.942
	RFE + PCA	95.1	0.973	0.94	0.962	0.951
SVM	Original	84.3	0.84	0.95	0.5	0.69
	RFE	90.2	0.913	0.89	0.90	0.90
	RFE + PCA	91.1	0.937	0.89	0.935	0.91
K-NN	Original	87.4	0.87	0.96	0.57	0.747
	RFE	87.6	0.98	0.77	0.99	0.874
	RFE + PCA	90.2	0.98	0.83	0.98	0.90
Bagging	Original	83.2	0.833	0.912	0.631	0.75
	RFE	89.8	0.972	0.879	0.935	0.898
	RFE + PCA	91.3	0.961	0.90	0.941	0.921
	RFE + PCA	91.3	0.961	0.90	0.941	0.921

RFE, Recursive Feature Elimination; PCA, Principle Component Analysis; SVM, support vector machine; K-NN, K-nearest neighbours’ algorithm.

**Table 4:** Hyperparameter settings of each ML classifier.

PD Dataset	Positive PD
MLP	Number of iterations = 500, learning rate = 0.01, Solver for optimum weight = adam
SVM	Regularization parameter = 1, kernel = rbf, Gamma = 2
K-NN	Number of neighbors = 1, Leaf size = 40
Bagging	Classifier = K-NN, Number of iterations = 100, max samples = 0.9

**Table 5:** Evaluation results based on the RFE + PCA models.

Classifier	Accuracy	Precision	Sensitivity	Specificity	G-Mean
SVM	98.2	0.99	0.97	0.99	0.98
MLP	96.4	0.97	0.94	0.97	0.96
Bagging	95.1	0.99	0.91	0.96	0.95
K-NN	93.8	0.98	0.94	0.99	0.94

**Table 6:** Comparison results between this study and other studies.

Study	Number of Features	Accuracy	Year of Publication
CNN method [8]	-	86.90	2019
MAMT method [36]	50	92.46	2020
XGBoost method [10]	21	84.80	2020
Random Forest method [11]	54	90.20	2021
Proposed	18	98.2	-

## Data Availability

Not applicable.
